# Assessing Effect Modification of Excess Winter Death by Causes of Death and Individual Characteristics in Zhejiang Province, China: A Multi-Community Case-Only Analysis

**DOI:** 10.3390/ijerph15081663

**Published:** 2018-08-06

**Authors:** Yiling He, Xuehai Zhang, Meng Ren, Junzhe Bao, Cunrui Huang, Shakoor Hajat, Adrian G Barnett

**Affiliations:** 1 School of Public Health, Sun Yat-sen University, Guangzhou 510080, China; heyling@mail2.sysu.edu.cn (Y.H.); melodyrren@163.com (M.R.); junzhe_bao@126.com (J.B.); 2 Zhejiang Provincial Center for Disease Control and Prevention, Hangzhou 31004, China; xhzhang@cdc.zj.cn; 3 Department of Social & Environmental Health Research, London School of Hygiene & Tropical Medicine, London, UK; Shakoor.hajat@lshtm.ac.uk; 4 School of Public Health and Institute of Health and Biomedical Innovation, Queensland University of Technology, Queensland 4059, Australia; a.barnett@qut.edu.au

**Keywords:** winter mortality, modify effect, individual characteristic, Cosinor model, case-only analysis

## Abstract

Mortality in many parts of the world has a seasonal pattern, with a marked excess of deaths during winter. To date, however, there is very little published evidence on the nature of this wintertime excess in low- and middle-income countries. In this study, we aimed to quantify the extent of the death peak in winter and to assess effect modification on excess winter death (EWD) by individual characteristics and cause of deaths in China. We used a Cosinor model to examine seasonal patterns for specific causes of deaths and a case-only analysis of deaths in winter compared with other seasons to assess effect modification by individual characteristics. A total of 398,529 deaths were investigated between January 2010 and December 2013 in Zhejiang Province, China. Deaths peaked in winter, and overall mortality was around 30% higher in winter than in summer. Although diseases of the respiratory and circulatory systems were highly seasonal, surprisingly we observed that deaths from mental and behavioral disorders exhibited greater fluctuation. Males, the elderly and illiterate individuals suffered high EWD. EWDs were also particularly common in emergency rooms, at home, on the way to hospitals, and in nursing homes/family wards. This study highlighted the high EWD in some previously unreported groups, indicating new information to facilitate the targeting of necessary preventive measures to those at greatest risk in order to mitigate wintertime death burdens.

## 1. Background

Despite long-term improvements in healthcare, housing and other factors, seasonal variation in mortality remains a problem in many parts of the world, with a marked excess of deaths still present during winter months [1]. Much of this excess winter mortality can be attributed to exposure to wintertime weather, especially ambient temperature; however, other factors such as seasonal infections are also important. A common and straightforward way to measure this wintertime excess is to calculate the Excess Winter Death (EWD) statistic. This metric is the ratio of deaths in the winter period compared with deaths in the non-winter period [2]. Although subject to interpretational limitations [3], the EWD value is a useful tool to quantify the overall health burden associated with the winter months and it is readily comparable between different populations. A population-based study in the UK showed a 30% increase in winter deaths [4], and similar winter increases have been found in other countries in Europe and also in the United States, Canada, Australia, New Zealand and Japan [5,6,7,8,9,10].

To date, however, there is very little published evidence on the nature of this wintertime excess in low- and middle-income countries [11]. EWD was explored in a study from Burkina Faso; however, this was based on only 4098 deaths [12]. Although the explicit relationships between temperature and mortality have now been characterized in China [13], evidence of EWD in Chinese populations is still very limited despite the large populations involved and the varied climatic and socio-economic conditions. The Ou et al. study reported a 26% wintertime excess in all-cause mortality in the subtropical city of Guangzhou, China [14]. Mortality records from China contain rich information on individual characteristics such as education level and place of death and so allow for the EWD metric to be calculated for important sub-groups of the population which can inform more targeted interventions. Previous studies did consider EWD modification by cause of death, sex, age, education and place of death, but categorizations were limited.

Evidence of EWD-modification by disease mainly relates to broad cause-of-death groups. Although influenza epidemics contribute to the winter increase in mortality, the effect is more general with variation in other respiratory, cardiovascular and cerebrovascular diseases [6]. The winter increase is apparent at all ages but is greatest in relative and absolute terms in elderly people [4]. Besides factors associated with biological and genetic considerations that have been linked with reduced health status, the health of a population is influenced by a large number of factors such as socioeconomic status, behavioral patterns and environmental parameters [7,15]. Some studies have also reported that EWD risk is greater in more deprived groups; however, these are usually based on area-level measures of deprivation [16]. Other factors such as place of death and more specific causes of death have generally not been fully explored in most settings around the world, usually because of power limitations. Assessment of the large populations of China allows us to address this research gap.

Understanding seasonal patterns in mortality and the underlying causes of excess winter mortality have clear implications for many aspects of public health as these could be used to guide targeted health-related interventions. In this study, we examined all-cause mortality in Zhejiang Province in China to quantify the extent to which deaths peak in winter and to explore differences according to the primary cause of death. We also examined the degree to which excess winter mortality is modified by individual socio-demographic characteristics. We hypothesize that some sub-groups of the population will exhibit greater EWD and thus should be the priority for public health intervention measures.

## 2. Methods 

### 2.1. Study Setting

Zhejiang is an eastern coastal province of China. It has a mainly subtropical monsoon climate, with a clear division of four seasons. Winters are often dry and cold with low temperatures and little rainfall; summers are usually wet and hot with high temperatures. The average annual temperature in Zhejiang Province is 15–18 °C, and its coldest and hottest days are usually seen in January and July, respectively. The province is divided into eleven prefecture-level divisions, and they are subdivided into 90 county-level divisions (32 districts, 22 county-level cities, 36 counties). Han Chinese people make up the vast majority of the population.

### 2.2. Data Source

Death data were collected from the Chinese National Disease Surveillance System. We obtained a total of 30 county-level surveillance sites across the whole Zhejiang Province from January 2010 to December 2013. The death certificate includes demographic and other important information, such as gender, nationality (race), marital status, education, date of death, date of birth, and place of death. Medical conditions are also listed on death certificates according to the International Classification of Diseases, 10th Revision (ICD-10). In China, either a medical practitioner or a coroner is required to certify the cause of death, and they should report on all underlying conditions that directly caused or contributed to the death.

### 2.3. Statistical Analysis

In a descriptive analysis, we plotted the monthly number of deaths in all regions by ICD chapter. The plots showed a strong annual seasonal pattern, and hence we examined the effect of season for specific causes using a Cosinor model and assumed a single annual cycle [17]. We fitted a separate model for each ICD chapter and adjusted for a linear trend over time. The Cosinor model estimates the increase in seasonal deaths (amplitude) and the location of the peak increase (phase). Due to low numbers of deaths (fewer than 50), we excluded the following ICD chapters for analysis: Diseases of the eye and adnexa; Diseases of the ear and mastoid process; and Pregnancy, childbirth and the puerperium.

We used a case-only analysis to identify individual characteristics that may increase the risk of dying during winter. This is a logistic regression model with winter death (yes/no) as the dependent variable and individual characteristics (e.g., age, gender, education) as the independent variables. We started with a model with all the available individual characteristics and then removed variables that appeared to be co-linear based on the variance inflation factor (VIF). We present the results as prevalence ratios and 95% confidence intervals (CIs) rather than odds ratios, as prevalence ratios are easier to interpret because they can be used as direct multipliers (e.g., twice as likely to die in winter) [18].

The case-only design was originally proposed to improve efficiency in the study of gene-environment interactions by examining the prevalence of a specific genotype only among cases. As Gouveia suggested [19], this approach can be used to investigate how individual characteristics that do not vary over time (e.g., gender or socioeconomic status) modify the effect of a time-varying exposure (e.g., season) on the outcome of interest by restricting the sample to cases (e.g., deaths). In our study, we applied this approach by comparing the individual characteristics of those dying in winter with those dying in other seasons. If an individual characteristic increases the risk of dying in winter, the proportion of deaths with that characteristic will be larger in winter. However, it should be noted that in the case-only design, a negative rate ratio does not necessarily indicate a decrease in the risk of dying for the group of individuals examined. Rather, this indicates that the increase in the risk of dying is less pronounced in winter for that group of individuals [20].

## 3. Results

**Figure 1:**
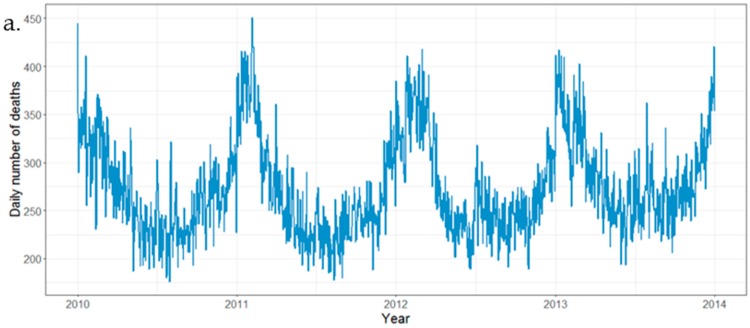

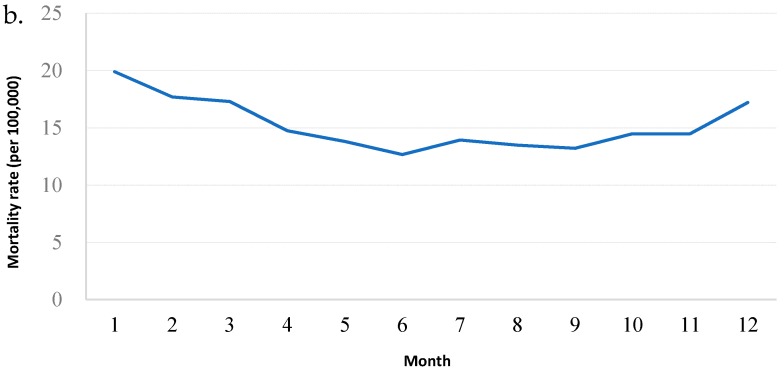
Summary statistics for death data, 2010–2013. (**a**). Time series of daily deaths; (**b**). Mortality rate (per 100,000 persons), 2010–2013. This was calculated based on the Chinese census in 2010.

During the four-year period of 2010–2013, there were 398,529 deaths registered in 30 counties in Zhejiang Province, with an average of 364 deaths per day (Table 1). Deaths peaked in the winter months of December, January and February, and were lower in the summer months of June, July and August, varying by 30% or more between the highest and the lowest months (Figure 1). The overall rate ratio between winter and non-winter months was 1.61 (60% winter increase), and the average number of deaths per day was 442 in winter and 275 in summer. There was little evidence that the ratio varied by geographical region.

Almost 80% of the deaths occurred at home, and approximately 15% of deaths were among people admitted to a hospital (both hospital ward and emergency room). Most deaths were among Han nationality (99.9%) who had no education (57%) (Table 1). More than 80% of deaths occurred among people aged 60 or over, and the median/mean age of death was 72 years old (70 for males and 75 for females).

**Figure 2:**
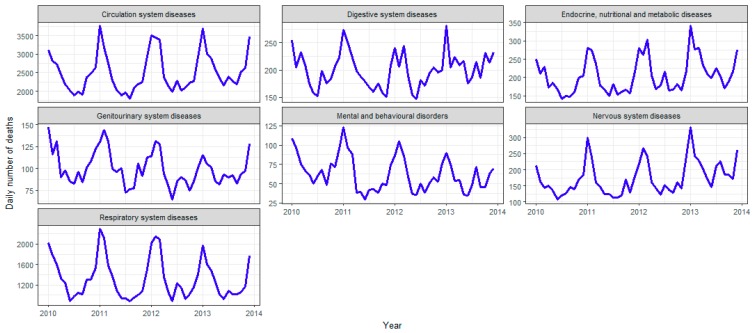
Time series of daily deaths by specific causes of death with seasonal pattern.

The leading underlying causes of death were circulatory diseases, neoplasms and respiratory diseases, which accounted for 75% of all deaths (Table 2). Many diseases appeared to have strong seasonal patterns with a winter peak, such as diseases of the circulatory system, respiratory system, nervous system, digestive system, genitourinary system, mental and behavioral disorders, and endocrine, nutritional and metabolic (Figure 2). Other causes, including external causes of morbidity and mortality, certain conditions originating in the perinatal period, diseases of the blood and blood-forming organs, did not display a clear seasonal pattern.

We found that the seasonal effect differs greatly for various causes of death (Table 3), and many causes are highly seasonal, including mental and behavioral disorders, diseases of the respiratory and circulatory system. However, a noticeable exception is cancer (although a large number of deaths were attributable to neoplasms, there was only a 2.5% increase in winter), which is almost equally spread amongst all seasons after removing the increasing trend (Figure 2 and Table 3). The results show that the seasonality of deaths is not inevitable for all persons who happen to be ill during winter. Instead, the mechanisms for increased winter mortality are specific to particular causes of death.

We compared winter deaths with deaths in other seasons using a case-only analysis. Based on a high VIF, we dropped marital status from the model. We found that males were slightly more susceptible during winter than females (Table 1). Those without education were also more likely to die in winter compared with other education groups. People who died in an emergency room, at home, on the way to hospital, in nursing home and family ward, were more likely to die in winter than those who died in a hospital ward. Deaths outside of the city were less likely in winter. Age was a strong predictor of winter death; young people were at a slightly increased risk, and the old were much more likely to die in winter with an increased risk above age 60.

## 4. Discussion

This study provides evidence for obvious seasonal mortality in Zhejiang Province, China, where deaths peaked in winter and overall mortality was around 30% higher in winter than summer. Deaths are predictable to some extent, in that they occur more often for particular causes such as cardiovascular and respiratory disease, mental and behavioral disorders. Individual characteristics such as age, gender, education are important predictors of seasonal mortality. Males, those older than 60 and those without education were more susceptible during winter. Also more common in winter were deaths in the emergency room, at home, on the way to hospital, in a nursing home and family ward.

The season of the year exerts an influence on total mortality, driven by a strong seasonal pattern in deaths due to circulatory and respiratory diseases. Mortality from respiratory disease was strongly influenced by season, with winter mortality estimates 35.9% higher than expected from non-winter seasons. As for the determinants of excess winter mortality from respiratory disease, cold weather is usually regarded as playing a dominant role. Other factors, such as influenza and other infections, are also involved in driving this kind of clear seasonal pattern [21].

Winter increases in circulatory disease mortality have been observed in other countries [19,22,23,24,25]. This was true in our study when deaths from circulatory disease had the largest absolute winter increase, as it comprised 30% of total mortality. Several explanations have been put forward, from misclassification of circulatory disease deaths [26] to changes in air temperature [27]. In addition, circulatory risk factors (e.g., waist circumference, blood pressure) showed a seasonal pattern characterized by higher levels in winter. This pattern could contribute to the seasonality of circulatory mortality [28], offering another potential explanation.

In our study, deaths attributable to mental and behavioral disorders had the strongest seasonal pattern (37.4% increase in winter), but this grouping contributed only 0.8% of total winter mortality. Previous studies on seasonality of bipolar disorder and depressive disorder showed a clear peak in the winter months [29,30,31]. A longitudinal study conducted in Ireland reported the highest levels of depressive symptoms in winter, and those who live in areas with longer daily hours of sunshine experienced lower occurrence rate of depressive symptoms [32]. In addition, the Kadotani et al. study revealed a higher rate of suicide attempts after a few days with lower levels of sunlight in Japan [33]. Similar associations were also found in Australia and in Austria [34,35]. However, studies in the Northern Hemisphere, including Ireland, England, and Scotland, have shown that the peak time of the first hospital entries for mental disorders occurs in summer [36,37]. It could be explained that most of them were conducted in developed populations where weather characteristics, socio-economic conditions, and culture differ from those in China. In addition, ambient temperature has long been suspected of being a cause of psychotic exacerbation in mental and behavioral disorders [38]. Hence, we speculate that low ambient temperature in winter may contribute to more frequent onset of mental and behavioral disorders exacerbation.

Analysis using ICD chapters may have limitations as these categories are broad and are not based strictly on etiology. For example, the Wilkinson et al. study [4] noted that respiratory disease seemed to be a strong determinant of cardiovascular but not respiratory death. A previous analysis of New Zealand deaths with infectious disease causes found that only 10% of those records were coded to the infectious disease chapter [39]. The specificity of association may have been obscured by misclassification of cause of death, but analyses of deaths with any mention of respiratory/infectious causes also did not show an association with pre-existing illness.

Healy argued that low socioeconomic status increased the risk of winter deaths in Europe [40]. Hales found that there was an increased risk of dying in winter for most New Zealanders, but more so among low-income people [7]. It also seems that in China, winter mortality is increased amongst people of lower socioeconomic status (e.g., illiteracy group, people died at home). There are some possible explanations, including poorer health status, scarce medical resources, and low indoor temperatures due to inadequate heating and household crowding. In a more recent study, education serving as a proxy for socioeconomic status has also been highlighted as a determinant for seasonal mortality patterns [40]. However, exact causal mechanisms are not known. Hence, further work is needed to disentangle the complex relationships between different indicators of housing quality and other measures of socioeconomic deprivation, and then to clarify their effects on seasonal mortality.

Previous studies have shown that females are more vulnerable than males to winter mortality. In the UK, women had a larger risk than men for reasons other than their greater age, health status, social isolation, or socioeconomic position [4]. In New Zealand, the risk for females was 9% higher than for males after adjusting for all major covariates [39]. The existing evidence seems to indicate that the lower capability of producing maximum heat vasoconstriction puts females at greater risk during cold spells [41,42,43]. Whereas there is an inverse result in China, although only a slight difference exists between genders. The higher risk of dying in winter among Chinese men does not seem to be due to clinic or socioeconomic differences, but we know that smoking rate in China is much higher for men than for women. Although it is not fully explained, this may imply that smoking could be an important lifestyle risk factor for higher winter mortality.

One hypothesis to explain these seasonal variations in mortality is that they simply represent shifts in the timing of events that lead to death. The effect of season could be seen as a kind of “harvesting (sometimes known as mortality displacement)” of vulnerable members of the population, such as elderly people. This view might be supported by the age gradients and causes of death, which demonstrate the greatest seasonal variation. A study in France showed a clear seasonal pattern both in pulmonary embolism (PE) incidence and mortality. In addition, the study supported an association of age on the PE seasonal variations [44]. However, it would require a detailed prospective study involving information about the pre-existing illness of those dying in different seasons to investigate this hypothesis further [6].

Previous studies have shown that excess winter deaths are higher in out-of-hospital mortality than in-hospital deaths [14,45]. As hospitals are well-equipped with heating equipment, people who lived in hospitals are less likely to be exposed to low ambient temperature conditions. That is a possible explanation for the lower winter increase in in-hospital mortality than out-of-hospital mortality. Our study also confirmed this; people are more likely to die during winter when they are at home, on the way to hospital, emergency home, family ward or nursing home than in hospital.

Other explanations for the observed seasonal patterns of mortality are due to particular characteristics of the environment, such as extremes of temperature and humidity, different exposure to either indoor or outdoor air pollution, changing patterns in the frequency of seasonally-variable infectious agents, and season-related changes in lifestyle (e.g., diet, physical activity). Specifically, cold weather has been reported to be associated with increased risk of death from cardiovascular and respiratory diseases [46,47,48].

Indoor air quality may be worse in winter due to the use of polluting indoor heaters. As a study in France showed, the nitrogen dioxide concentration in indoor air particularly increased only during winter and was well correlated with the formic acid concentration [49]. In addition, inadequate and poorly designed ventilation in crowded places may boost exposure to air-borne pathogens by increasing their concentration in stagnant air and by re-circulating contaminated air. Furthermore, low ventilation rates were associated with increased behavior problems and health symptoms [50].

Although much of the winter excess seems to be related to cold, some countries have a larger seasonal fluctuation in mortality than others despite having milder winters [51]. Different from cold-related mortality, excess winter deaths reflect excess mortality during winter when compared with other seasons, and are influenced by low temperature, individual characteristics and potential diseases. Thus, concentrating solely on cold-driven deaths is far from reducing the high death rate during the winter season. Heating measures and adjusting indoor temperature should go along with identifying and protecting populations who are vulnerable to winter season. Hence, understanding the significance of EWD has a fundamental public health importance.

Exploring the effects modification by meteorological variables (e.g., temperature, humidity) or confounders with other seasonal exposures (e.g., air pollution, influenza) on EWD was beyond the scope of this research. Further research should explore more detailed or broader range of pathways, so that more targeted interventions can be achieved. For example, if EWD were associated with low indoor temperatures, campaigns to reduce winter mortality need to enhance protection against outdoor as well as indoor chill. If there exists a link between winter death peaks and influenza epidemics, then greater emphasis might be placed on more comprehensive influenza vaccination programme. Future research could also explore other seasonal exposures that may have potential to simulate EWD, such as air pollution, behavior patterns and household crowding [39].

This research has provided rare evidence on individual characteristics of risk for winter deaths in China. Since winter increases are predictable for specific causes of disease and vulnerable population groups, public health measures might play a part. The fact that the risk of dying in winter is far greater in the elderly suggests that additional measures are needed to reach all those at risk.

## 5. Conclusions

Increased winter mortality is an important public health problem in China. Investigating seasonal patterns of death can help explain changes in the health of a population, and provide insight into risk factors that contribute to the burden of higher winter mortality. Death data are particularly useful for understanding the health of the overall population, investigating differences in the patterns between population groups, and informing health policy, planning, investment and administration of the health care system.

## Figures and Tables

**Table 1:** Summary statistics for total deaths (2010–2013), prevalence ratios and 95% CIs for winter deaths compared with deaths in other seasons ^a^.

Individual Characteristics	Deaths	Prevalence Ratios	95% CIs
Age			
0–17 years	4880 (1.2%)	1.00	
18–44 years	16,526 (4.1%)	1.08	1.00–1.16
45–59 years	47,364 (11.9%)	1.03	0.96–1.10
60–79 years	157,310 (39.5%)	1.12 *	1.05–1.19
> 80 years	172,449 (43.3%)	1.30 *	1.22–1.39
Gender			
Male	227,100 (57.0%)	1.00	
Female	171,429 (43.0%)	0.99 *	0.97–1.00
Education			
Illiteracy	227,038 (57.0%)	1.00	
Primary school	111,604 (28.0%)	0.93 *	0.91–0.95
High school	51,714 (13.0%)	0.92 *	0.89–0.94
University	6551 (1.6%)	0.94 *	0.88–0.99
Not clear	1622 (0.4%)	0.95	0.85–1.06
Ethnicity			
Han	398,194 (99.9%)	1.00	
Others	335 (0.1%)	0.88	0.72–1.08
Death place			
Hospital	46,911 (11.8%)	1.00	
Home	316,668 (79.5%)	1.11 *	1.06–1.16
On the way to hospital	13,073 (3.3%)	1.12 *	1.09–1.14
Emergency room	11,597 (2.9%)	1.06 *	1.02–1.11
Outside the city	6137 (1.5%)	0.87 *	0.82–0.93
Family ward/Nursing home	2081 (0.5%)	1.19 *	1.08–1.31
Others/Not clear	2062 (0.5%)	0.91	0.82–1.00
Total	398,529		

^a^: We used the variance inflation factor (VIF) to judge if variables were co-linear. Based on a high VIF, we dropped marital status from the model; *: *p* < 0.05.

**Table 2:** Causes of death by ICD chapter.

#	ICD Chapter	Number	Percentage
1	Certain infectious and parasitic diseases	5882	1.5
2	Neoplasms	115,324	28.9
3	Diseases of the blood and blood-forming organs and certain disorders involving the immune mechanism	791	0.2
4	Endocrine, nutritional and metabolic diseases	9865	2.5
5	Mental and behavioural disorders	3013	0.8
6	Diseases of the nervous system	8391	2.1
7	Diseases of the eye and adnexa	37	0.0
8	Diseases of the ear and mastoid process	8	0.0
9	Diseases of the circulatory system	120,544	30.2
10	Diseases of the respiratory system	63,589	16.0
11	Diseases of the digestive system	9613	2.4
12	Diseases of the skin and subcutaneous tissue	697	0.2
13	Diseases of the musculoskeletal system and connective tissue	1905	0.5
14	Diseases of the genitourinary system	4797	1.2
15	Pregnancy, childbirth and the puerperium	34	0.0
16	Certain conditions originating in the perinatal period	1029	0.2
17	Congenital malformations, deformations and chromosomal abnormalities	1290	0.3
18	Symptoms, signs and abnormal clinical and laboratory findings, not elsewhere classified	12,594	3.2
20	External causes of morbidity and mortality	39,126	9.8
	Total	398,529	100.0

There is no death attributed to ICD Chapters #19, #21 and #22 in the dataset.

**Table 3:** Seasonal patterns in deaths by ICD chapter.

ICD Chapter	Number of Deaths	Growth Rate (%)	Date
Certain infectious and parasitic diseases	15.6 *	12.7	17 January
Neoplasms	59.8 *	2.5	17 December
Diseases of the blood and blood-forming organs and certain disorders involving the immune mechanism	2.4 *	14.9	13 November
Endocrine, nutritional and metabolic diseases	53.2 *	25.9	1 February
Mental and behavioural disorders	23.5 *	37.4	29 December
Diseases of the nervous system	51.0 *	29.3	13 January
Diseases of the circulatory system	644.4 *	25.7	13 January
Diseases of the respiratory system	475.9 *	35.9	20 January
Diseases of the digestive system	32.4 *	16.2	13 January
Diseases of the skin and subcutaneous tissue	2.7 *	18.3	29 January
Diseases of the musculoskeletal system and connective tissue	8.0 *	20.3	10 January
Diseases of the genitourinary system	20.8 *	20.8	13 January
Certain conditions originating in the perinatal period	1.5	7.2	28 September
Congenital malformations, deformations and chromosomal abnormalities	3.3 *	12.3	12 February
Symptoms, signs and abnormal clinical and laboratory findings, not elsewhere classified	84.9 *	32.4	4 January
External causes of morbidity and mortality	34.3	4.2	29 December
Total	1495.8	18.0	13 January

The number of deaths is the peak increase in absolute number of deaths, which means the number of deaths attributed to certain diseases. The growth rate is the percent increase from the year-round average. For example, the number of deaths attributed to ICD-1 (Certain infectious and parasitic diseases) is 15.6 per day with a phase of 17 January. ICD Chapters #7, #8 and #15 (i.e., Diseases of the eye and adnexa; Diseases of the ear and mastoid process; Pregnancy, childbirth and the puerperium) were excluded due to a low number of deaths (under 50); *: *p* < 0.05.
